# Antibiotic susceptibility of *Escherichia coli* is affected by evolutionary history but not by history of elemental limitation

**DOI:** 10.1128/msphere.00538-25

**Published:** 2026-03-23

**Authors:** Marissa A. Donofrio, Heather L. Blasius, Catherine C. Nguyen, Alexa L. Schnell, Caroline B. Turner

**Affiliations:** 1 Biology Department, Loyola University Chicago 2456, Chicago, Illinois, USA; University of Nebraska Medical Center College of Medicine, Omaha, Nebraska, USA

**Keywords:** nutrient limitation, intrinsic resistance, *Escherichia coli*, experimental evolution

## Abstract

Antibiotic resistance in bacteria is a global public health threat. To understand how the evolution of antibiotic susceptibility is affected by environmental conditions and prior evolutionary history, we worked with populations from the Long-Term Evolution Experiment (LTEE) with *Escherichia coli*. These populations previously evolved independently for 50,000 generations in an environment without antibiotics, making them an ideal system for studying the effect of evolutionary history on adaptation to new selective pressures. We further evolved five of the LTEE populations, as well as their shared ancestor, under either carbon- or nitrogen-limited conditions and then tested intrinsic resistance to four antibiotics. Evolution under elemental limitation did not have a significant impact on resistance to any of the tested antibiotics. However, some LTEE populations did have higher resistance than other populations. Susceptibility also varied within one population, which had the lowest level of resistance to all four antibiotics. We hypothesized that resistance levels might differ between two clades of bacteria that have coexisted within this population for more than 40,000 generations. Interestingly, although antibiotic susceptibility varied within the population, there was no consistent difference between clades. Instead, one particular clone isolated from the population exhibited higher resistance than the other clones sampled. These findings indicate that antibiotic resistance can vary both within and between experimentally evolved populations, even in the absence of direct selection on resistance. Our results also show that measured levels of susceptibility may depend on stochastic sampling effects during isolation of clones.

**IMPORTANCE:**

Antibiotic resistance is one of the most pressing health challenges worldwide, and understanding how bacteria evolve resistance, even when not directly exposed to antibiotics, is critical for managing and predicting emerging threats. Our study leverages the unique Long-Term Evolution Experiment with *Escherichia coli* to show that both the evolutionary history of bacterial populations and random variation among individual clones can significantly influence intrinsic antibiotic susceptibility. Our results also suggest that elemental limitation, while a critical environmental variable, may not be an important driver of intrinsic antibiotic susceptibility, at least over short time frames.

## INTRODUCTION

Although clinical antibiotic resistance most often occurs as an evolutionary response to antibiotic exposure, bacteria also vary in intrinsic resistance prior to antibiotic exposure (1). Intrinsic resistance levels are typically lower than clinical resistance, yet even low levels of resistance can promote subsequent evolution of higher resistance levels (2, 3). Moreover, intrinsic resistance can evolve as a pleiotropic consequence of other rapidly evolving characteristics such as heat stress sensitivity (4), membrane permeability (5), biofilm production (6), and expression levels of transporters and porins (7). Because intrinsic resistance is influenced by a large number of genes (8–10), such pleiotropic interactions are especially likely. Here, we sought to understand how environmental variation, specifically competition for scarce elemental nutrients (carbon and nitrogen), shapes the evolution of intrinsic resistance.

Elemental limitation (often referred to as nutrient limitation) is a key environmental variable that underlies the evolution of many conserved traits. Depending on the environment, the population size of a species might be limited by the availability of different elements, including carbon, nitrogen, phosphorus, or iron (11–14). Productivity in aquatic and terrestrial ecosystems is most commonly nitrogen or phosphorus limited (15, 16). Elemental limitation can also be important in host-associated environments. For example, some hosts use restriction of elements such as iron to limit microbial growth (17, 18). The gut microbiome may be nitrogen limited (19), although elemental limitation in the gut is not well studied and may well vary between individuals and over time (20). The urinary tract, where *Escherichia coli* are a common cause of urinary tract infections, is nitrogen rich and likely carbon limited (21).

Despite the extensive study of elemental limitation as an environmental characteristic, little is known about the effects of elemental limitation on the evolution of intrinsic antibiotic resistance. Several studies have found that bacteria are more antibiotic sensitive under conditions with excess carbon in the form of glucose or other sugars (22, 23). However, those studies focused on physiological, rather than evolutionary, responses to resource availability. A few studies provide indirect evidence for evolutionary interactions between elemental limitation and antibiotic resistance. For example, selection for tobramycin resistance results in mutations in a protein in a regulatory system that responds to nitrogen availability in both *Pseudomonas aeruginosa* and *Acinetobacter baumannii* (24, 25), suggesting potential interactions between nitrogen-limitation responses and tobramycin resistance in these species.

In the Long-Term Evolution Experiment (LTEE) with *E. coli*, 12 populations have evolved in the absence of antibiotics under carbon-limited conditions for tens of thousands of generations (26, 27). The evolved LTEE bacteria were typically more susceptible to antibiotics than ancestors, with the largest changes occurring during the first 2,000 generations of evolution (28, 29). Evolved strains were more sensitive to both tetracycline and ampicillin, which arose solely as pleiotropic effects of selection on other traits. However, the direction of pleiotropic effects on intrinsic resistance can vary depending on the particular organism and environment. For example, in an evolution experiment with *E. coli* and *Salmonella*, Knöppel et al. (30) found more cases of increased resistance than decreased resistance. The pleiotropic effect can also depend on the specific bacterial mutation and the antibiotic tested (31).

Different populations that evolved in the same environment can also differ in their resistance levels, even if all populations evolved from the same ancestor. These differences constitute the role of evolutionary history (32, 33). The long time period of the LTEE provides the opportunity to test the effects of evolutionary history on intrinsic resistance. The populations have diverged in many characteristics not under selection, such as ability to grow on resources not present in the experiment (34, 35) and fitness in an anoxic environment (36), suggesting that they may also have diverged in intrinsic antibiotic susceptibility and in evolutionary response to elemental limitation.

**Fig 1:**
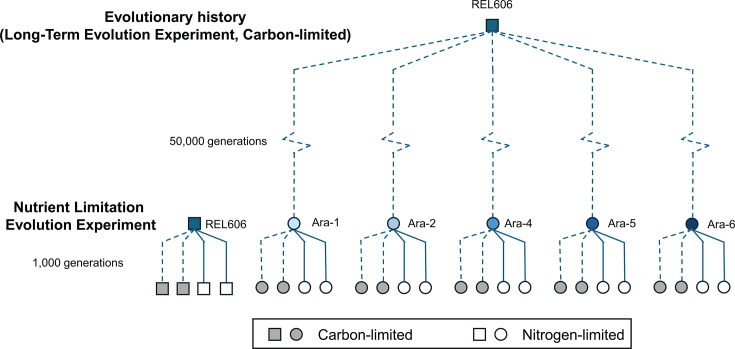
The populations in the elemental-limitation evolution experiment were founded from ancestral (REL606) and 50,000-generation evolved clones (Ara-1, Ara-2, Ara-4, Ara-5, and Ara-6) from the Long-Term Evolution Experiment. From each founding clone, two populations were propagated for 1,000 generations under carbon limitation and two under nitrogen limitation. Squares indicate the ancestor, REL606, and populations evolved for 1,000 generations from REL606. Circles indicate evolved clones from the Long-Term Evolution Experiment, and populations further evolved from those clones.

To test the effect of evolutionary history and elemental limitation on antibiotic susceptibility, we propagated *E. coli* strains isolated from the LTEE in either carbon- or nitrogen-limited media (Fig. 1). We found that strains descended from the same ancestor tended to have similar resistance to antibiotics, while strains descended from different ancestors may have varying resistance. However, there were no differences in resistance between strains evolved under carbon limitation or nitrogen limitation. This suggests that evolutionary history, but not elemental limitation, impacts intrinsic antibiotic susceptibility, at least over the timescale of our experiment.

## MATERIALS AND METHODS

### Evolution experiment

To study the impact of elemental limitation on intrinsic antibiotic susceptibility, we propagated clones from the LTEE (26, 37) in either carbon- or nitrogen-limited media. The founding strain of the LTEE is streptomycin resistant due to prior laboratory selection but has no known other antibiotic exposures and was derived from laboratory strains that were isolated before the clinical use of antibiotics (38, 39). We propagated 24 populations, 4 populations founded from each of 6 clones from the LTEE, for 1,000 generations (151 days). For each set of four, we propagated two replicate populations under carbon limitation and two under nitrogen limitation (Fig. 1; Table S1). The founding strains included clones from the 50,000-generation timepoint of five different LTEE populations as well as the ancestral strain of the LTEE populations, REL606 (Fig. 1; Table S1). The 50,000-generation clones we used were isolated from the Ara-1, Ara-2, Ara-4, Ara-5, and Ara-6 populations of the LTEE. We excluded population Ara-3, in which the ability to consume citrate evolved (40, 41), because its access to this additional carbon source in the Davis-Mingioli medium meant that it experienced different elemental supply ratios from the other populations (27). Of the populations we studied, Ara-1, Ara-2, and Ara-4 have evolved an elevated mutation rate, while Ara-5 and Ara-6 retain the ancestral mutation rate (37).

The 12 carbon-limited populations were grown under the same conditions as the LTEE, using Davis-Mingioli minimal medium with 25 mg/L glucose and 1,000 mg/L ammonium sulfate (26). The conditions for the 12 nitrogen-limited populations were identical except that the nitrogen-limited medium contained 250 mg/L glucose and 20 mg/L ammonium sulfate. We confirmed that the population size in this medium was limited by nitrogen availability (Fig. S1). The concentration of nitrogen was such that the population density was similar under carbon limitation and nitrogen limitation (~5 × 10^7^ cfu/mL). Populations were transferred daily via a 1:100 dilution. After 1,000 generations of evolution, we isolated a clone from each population for further analysis.

### Fitness measurements

We measured the fitness of all ancestral and evolved clones relative to REL607, the Ara^+^ mutant of REL606. The mutation conferring the ability to use arabinose serves as a neutral marker under the conditions of the LTEE (26). We used a paired sampling design, in which we compared each evolved clone with its ancestor. In all cases, we measured traits in the medium in which the derived clone had evolved. To acclimate the cells to those conditions, samples of frozen clones were first grown in Luria-Bertani medium, then transferred via 1:10,000 dilution into either nitrogen- or carbon-limited medium and incubated for 24 h. Other than the medium, conditions were the same as in the LTEE. Following the acclimation steps, equal culture volumes of REL607 and either an evolved or ancestral clone were added to fresh medium to start the competition assay. The population density of each competitor was measured by plating a diluted sample of the culture onto tetrazolium arabinose plates at the start and again after 24 h of competition. Tetrazolium arabinose plates allow differentiation of Ara^+^ and Ara^–^ competitors by colony color. We first calculated the fitness of the competitor (A) relative to REL607 (26) from the population sizes of each competitor before and after competition:


$$\frac{\log_{2}\left(\frac{A\left(Day=1\right)}{A\;\left(Day=0\right)}\right)}{\log_{2}\left(\frac{REL607\left(Day=1\right)}{REL607\left(Day=0\right)}\right)}.$$

Next, we calculated the change in fitness during our 1,000 generation experiment as the percent difference between the fitness of each derived clone and its proximate ancestor (either REL606 or a 50,000 generation evolved clone).

### Antibiotic susceptibility

To test for differences in antibiotic susceptibility in the evolved clones, we measured the minimum inhibitory concentration (MIC) for four different antibiotics. We chose antibiotics from a range of classes: ampicillin (beta-lactam), erythromycin (macrolide), tetracycline (tetracycline), and tobramycin (aminoglycoside). Clones were revived and acclimated as described for the fitness assays above. After 24 h of acclimation, we diluted the bacteria 1:100 into Mueller-Hinton broth with antibiotic. Inoculum size ranged from 10^7^ to 6 × 10^7^ cfu/mL. Thus, any measured differences in MIC reflect evolved differences in population size as well as differences in physiology. We measured the MIC in 96-well plates with Mueller-Hinton broth using twofold increases in antibiotic concentration. The MIC was recorded as the minimum concentration where no growth was visually observed following 24 h of static incubation at 37°C. We measured the MIC of all clones in Mueller-Hinton broth, rather than in their evolutionary environments, to provide a consistent measurement environment for all samples. We were unable to measure MIC in the evolutionary environments because population sizes in those environments were too low to allow accurate measurements of MIC.

For each antibiotic, we tested for significant effects of elemental limitation and founding clone on MIC with a non-parametric aligned rank transform analysis of variance (ANOVA) (42, 43) using the ARTool package in R v4.4.2 (44). We corrected for multiple comparisons using the Benjamini-Hochberg correction (45).

## RESULTS

### Fitness improved in the nitrogen-limited environment

**Fig 2:**
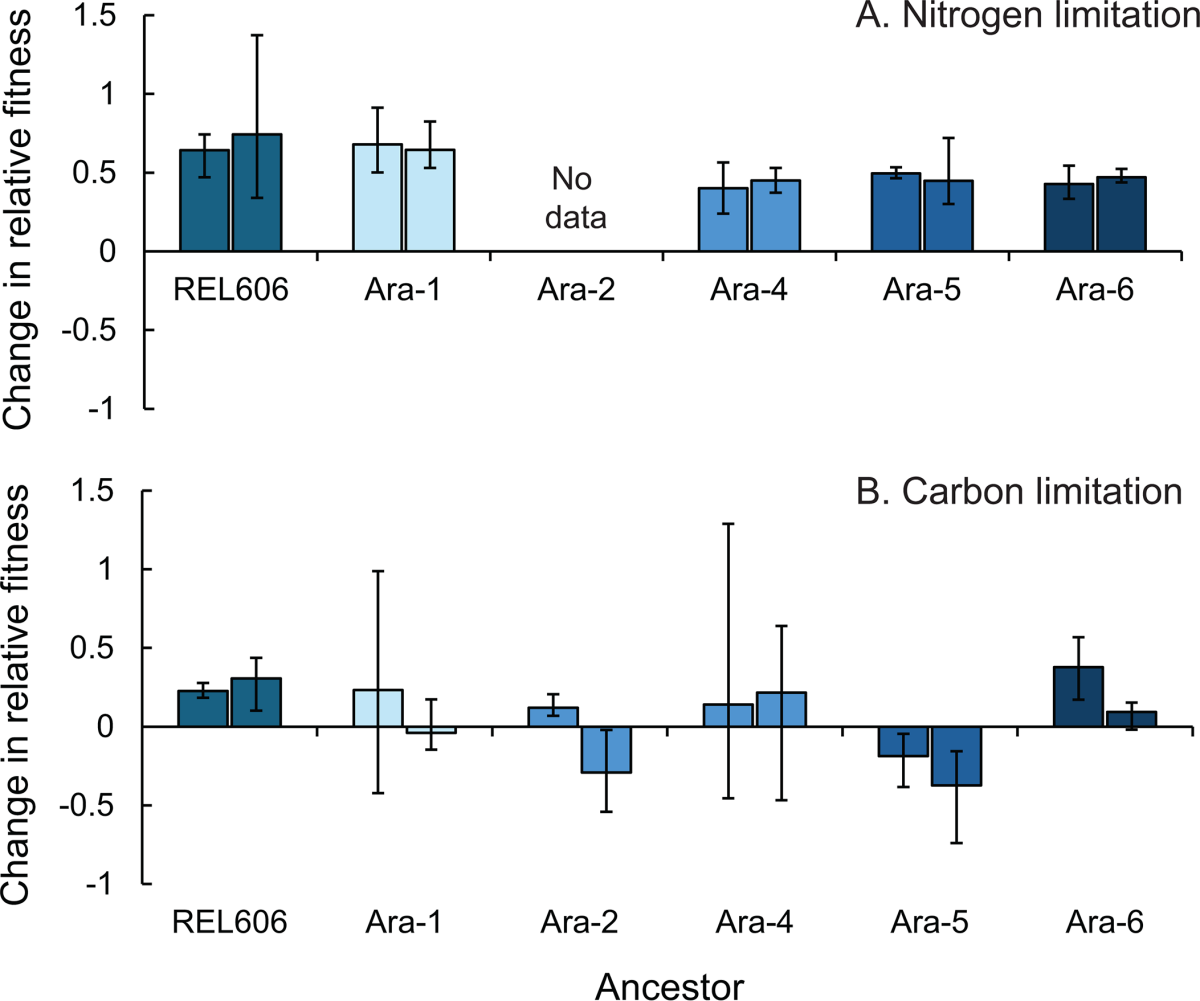
Fitness increased in nitrogen-limited but not carbon-limited environments. Each bar represents the percent change in fitness for a clone isolated from an evolved population compared to its ancestor. All evolved clones were assayed in their evolutionary environment. Percent change was calculated from the mean relative fitness across three paired replicate competitions against a common competitor (REL607). Fitness under nitrogen limitation increased significantly (paired *t*-test, *P* < 10^−6^) following 1,000 generations of evolution in a nitrogen-limited environment (**A**). Fitness under carbon limitation did not change significantly (*P* = 0.173) following 1,000 generations of evolution in the same carbon-limited environment as the LTEE (**B**). Error bars show the range of the three replicate measurements for each population.

Clones that evolved under nitrogen limitation were significantly more fit than their ancestors, indicating substantial genetic adaptation to the nitrogen-limited environment (Fig. 2A, paired *t*-test *P* < 10^−6^). After 1,000 generations of selection under nitrogen limitation, the evolved clones had a 56% higher fitness compared to their proximate ancestors. This represents a rapid gain in fitness, given that it took at least 20,000 generations for the populations in the LTEE to make similar fitness gains in a carbon-limited environment (46).

We were unable to directly measure fitness under nitrogen limitation for the clones derived from the 50,000-generation Ara-2 population of the LTEE because that clone and its descendants did not consistently produce colonies on tetrazolium arabinose agar after growing under nitrogen limitation. However, the common competitor underwent more generations of growth when it competed against the ancestral clone than when it competed against the evolved clones from those two populations. This strongly suggests that the Ara-2-derived populations also increased fitness under nitrogen limitation (see Supplemental text for details).

Fitness did not increase significantly in carbon-limited populations (Fig. 2B, paired *t*-test *P* = 0.173). This outcome is unsurprising given that 10 of these populations evolved from ancestors that had already evolved under carbon limitation for 50,000 generations in the LTEE. For these 10 populations, the average fitness change was 0.03. For the two populations derived from the ancestor that had not previously evolved in the carbon-limited LTEE (REL606), the mean fitness gain was much larger at 0.27. The large error bars for the carbon-limited competitions reflect the large fitness differences between the ancestor and clones derived from the 50,000-generation evolved ancestor populations. This fitness difference results in fewer countable ancestral colonies and therefore more variability in fitness measurements.

### Effects of evolutionary history on antibiotic susceptibility

**Fig 3:**
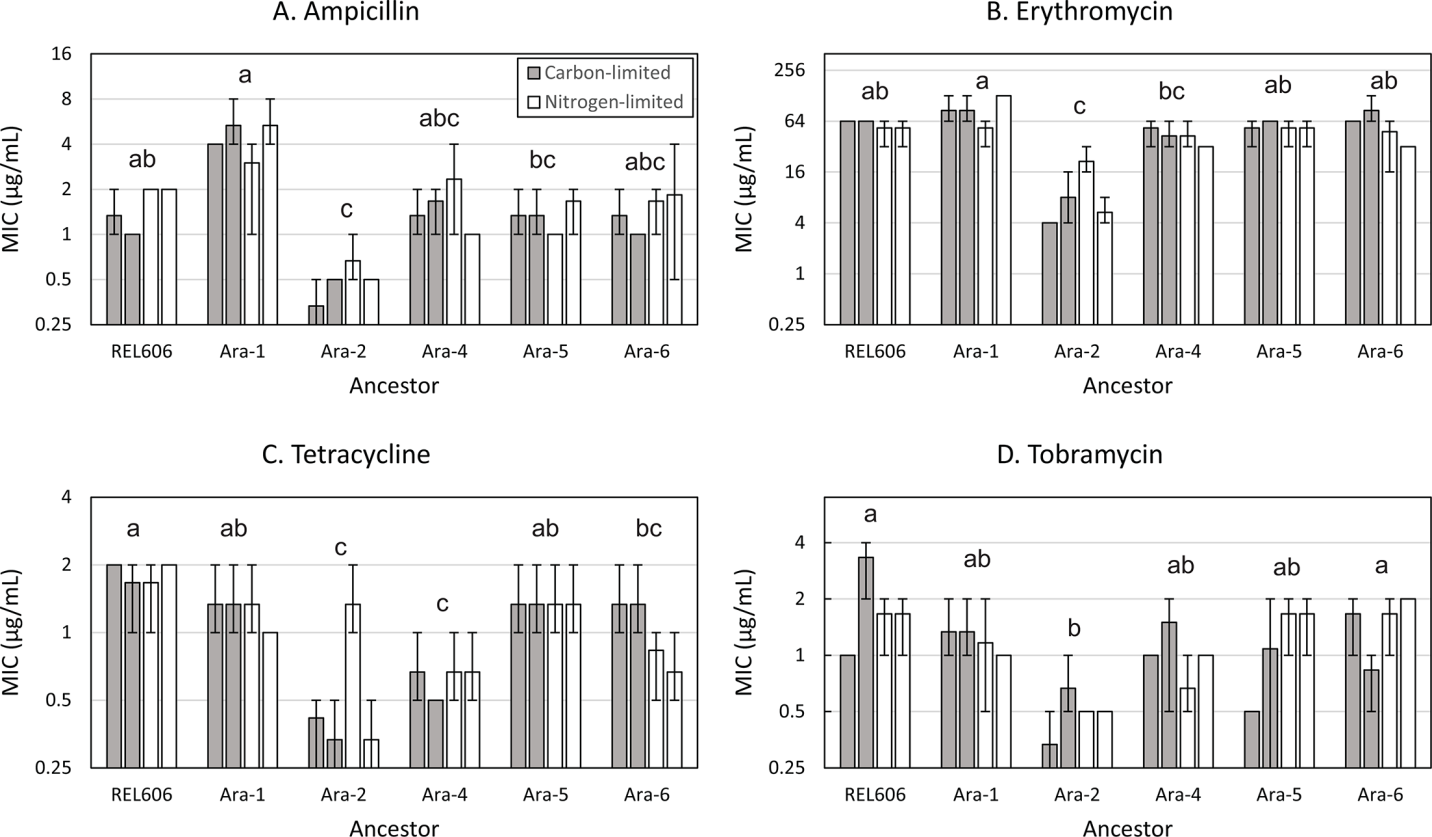
Minimum inhibitory concentration of ampicillin (**A**), erythromycin (**B**), tetracycline (**C**), and tobramycin (**D**) did not differ between carbon-evolved (black bar) and nitrogen-evolved (white bar) clones. Clones derived from the LTEE population Ara-2 had lower MICs than clones derived from other ancestors. Each bar represents the mean of three replicate MIC measurements. Error bars indicate the range of the replicate values. Groups that do not share a letter are significantly different from each other.

After correction for multiple comparisons, evolution under elemental limitation did not significantly affect MIC for any of the four antibiotics (Fig. 3; Table 1). The interaction between elemental limitation and evolutionary history was also not significant. However, prior evolutionary history did have a significant impact on MIC for all four antibiotics (Fig. 3; Table 1). Most strikingly, strains descended from Ara-2 were consistently among the least resistant strains compared to those derived from other ancestors. Ara-1- and REL606-derived strains were typically among the most resistant strains.

**TABLE 1:** *P*-values for non-parametric aligned rank transform ANOVA testing for the effect of ancestor (evolutionary history) and elemental limitation on the MIC for each antibiotic^*a*^

Drug	Ancestor	Elemental limitation	Ancestor:element interaction
Ampicillin	**0.002**	0.22	0.52
Erythromycin	**0.002**	0.04	0.18
Tetracycline	**3.4 × 10^−5^**	0.52	0.11
Tobramycin	**0.03**	0.74	0.10

^*a*^ Bold indicates terms that are significant after Benjamini-Hochberg correction for multiple comparisons.

### Variation in susceptibility within the Ara-2 population

We decided to further investigate the pattern of lower MICs for clones derived from the LTEE Ara-2 population. Surprisingly, in a previous analysis of susceptibility of LTEE strains to a wide range of antibiotics, Ara-2 MIC values were not noticeably different from other populations (29). We hypothesized that the difference between our results and those of Lamrabet et al. (29) was due to sampling of different lineages within the Ara-2 population, since the Ara-2 population contains two distinct clades that have maintained a long-term stable co-existence (47, 48). The two clades are easily identifiable because one clade produces large colonies on agar plates while the other produces small colonies. The Ara-2-derived populations in our evolution experiment were founded from a clone from the large-colony-forming clade (REL11333), while Lamrabet et al. (29) measured resistance of a small-colony-forming clone (REL11335). To quantify variation in resistance within the Ara-2 population, we isolated three additional clones from each of the two co-existing clades from the 50,000-generation Ara-2 population. We then measured the MIC of these clones, along with the clone we used from Ara-2 (REL11333) and the clone used by Lamrabet et al. (REL11335), following the same procedures as described above.

**Fig 4:**
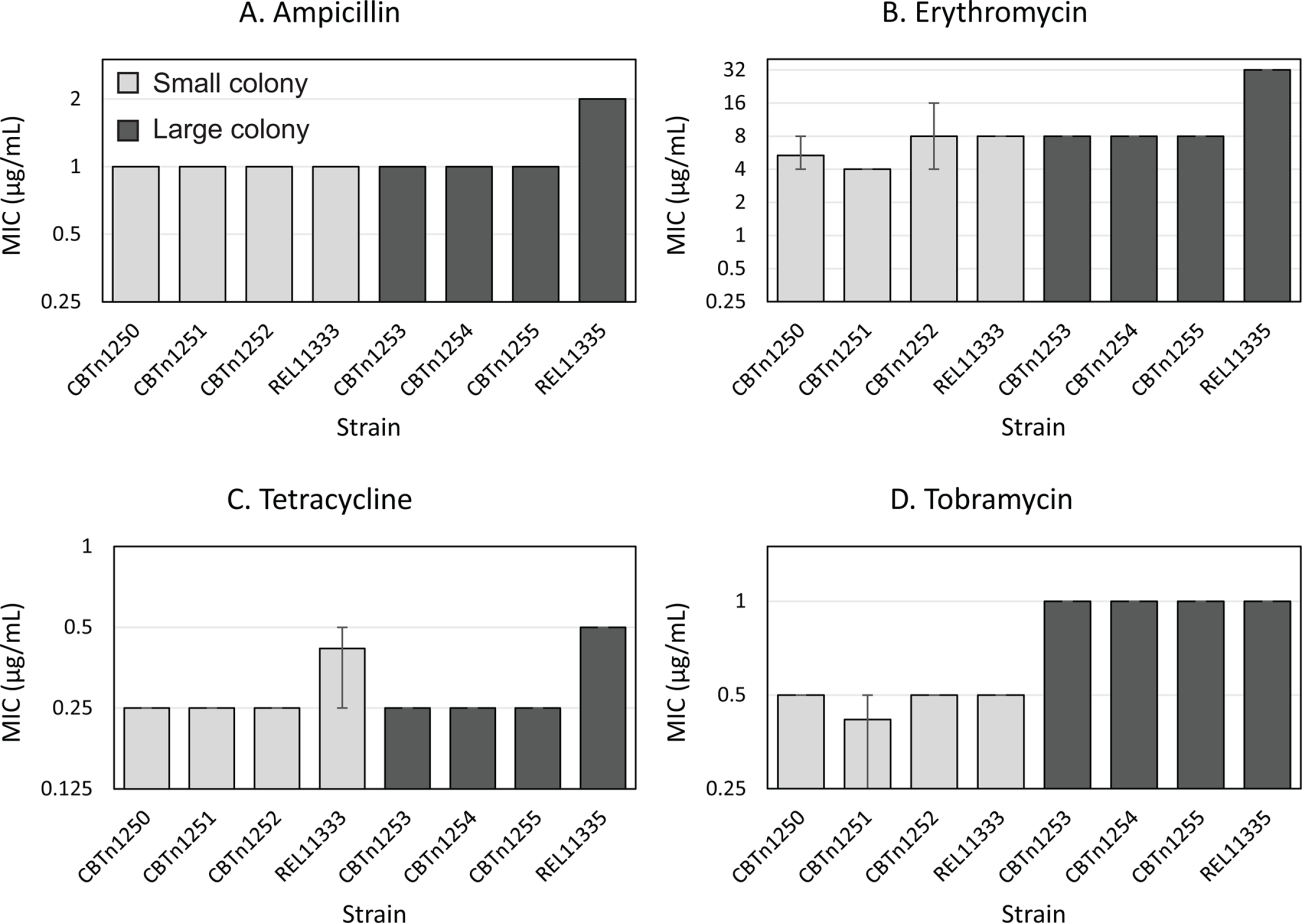
Minimum inhibitory concentration of (**A**) ampicillin, (**B**) erythromycin, (**C**) tetracycline, and (**D**) tobramycin did not differ between the small-colony-forming (light gray bars) and large-colony-forming (dark gray bars) clades of Ara-2. However, resistance was consistently high in REL11335, the clone used in Lamrabet et al. (29). Each bar represents the mean of three replicate MIC measurements. Error bars indicate the range of the replicate values.

There were no significant differences in ampicillin (non-parametric aligned rank transform ANOVA, *P* = 0.35), erythromycin (*P* = 0.10), or tetracycline (*P* = 0.87) resistance between large and small isolated Ara-2 clones, although large colonies were more tobramycin resistant than small colonies (*P* = 2 × 10^−4^; Fig. 4). However, the strain used by Lamrabet et al. (REL11335) was overall more resistant than all other Ara-2 clones (*P* < 1 × 10^−15^), suggesting that it was a single outlier and that typical Ara-2 strains were more susceptible to antibiotics than strains descended from other LTEE populations.

## DISCUSSION

Our experiment was designed to study the effects of elemental limitation and prior evolutionary history on intrinsic antibiotic susceptibility. We measured resistance to four antibiotics in clones derived from a 50,000-generation evolution experiment and then further evolved under either carbon or nitrogen limitation. Our results suggest that initial adaptation to low-nitrogen conditions did not have an effect on antibiotic susceptibility (Fig. 3; Table 1). Thus, there may not be systematic differences in intrinsic susceptibility between bacteria adapted to nitrogen-limited environments and those adapted to carbon-limited environments. There are two possible explanations for this response: either resistance changed by the same degree in both environments or resistance did not change in either environment. Because we did not measure the antibiotic resistance of the ancestral clones, we are unable to differentiate between these possibilities. The timeframe of our study was relatively short, but it was sufficient to allow for substantial adaptation to the nitrogen-limited environment (Fig. 2). It is possible that differences in susceptibility between carbon- and nitrogen-limited environments might emerge over a longer time frame or that more subtle differences in susceptibility could be detected with increased replication.

Antibiotic susceptibility did differ between clones descended from different populations of the LTEE (Fig. 3; Table 1). These populations had evolved independently for 50,000 generations in identical carbon-limited environments and from identical ancestors (26, 46). Thus, the observed variation in intrinsic resistance was due to the effect of history—chance variation in the mutations that occurred and spread in each LTEE population (32). Erythromycin sensitivity varied nearly eightfold between populations, indicating that chance variation can drive meaningful differences in intrinsic resistance. For the other antibiotics, the magnitude of the differences between populations was relatively small, on the order of a twofold change in MIC.

Previous research showed that LTEE populations differ in their ability to evolve resistance in response to antibiotic exposure and in the genetic basis of their evolved resistance (28, 49). Our results indicate that, in addition to varying in the evolvability of resistance, LTEE populations vary in their intrinsic susceptibility to antibiotics. In our data, resistance was generally greater in strains derived from the ancestor of the LTEE, consistent with previous findings that resistance declined over time in the LTEE (28, 29). Also consistent with the findings of Lamrabet et al. (29), there was no indication that resistance levels were related to mutation rate; the population with the highest MIC values (Ara-1) and the lowest MIC values (Ara-2) both have an elevated mutation rate.

For one particular population, Ara-2, we observed different patterns of resistance evolution than those observed previously (29). In our results, but not those of Lamrabet et al. (29), clones derived from the Ara-2 LTEE population had consistently lower levels of resistance compared to those from other LTEE populations. We suspected that the differences between our experiments could reflect within-population variation in antibiotic susceptibility in population Ara-2: Lamrabet et al. (29) used Ara-2 clone REL11335, while we used Ara-2 clone REL11333 as the ancestor to the populations in our evolution experiment.

Ara-2 has previously been well characterized in terms of its population diversity. Two lineages coexisted within Ara-2 for more than 50,000 generations (47, 50). These lineages, one which produces small colonies when plated and another which forms large colonies, coexist through a negative-frequency-dependent relationship based on cross-feeding of acetate (47, 48). We initially hypothesized that the variation in antibiotic susceptibility in Ara-2 would correspond to those two major clades. However, with the exception of a modest difference in tobramycin resistance, there was little overall difference in resistance between clones from the two clades (Fig. 4). Instead, most genotypes within population Ara-2 exhibited relatively low levels of resistance to the four antibiotics that we tested, but one genotype, REL11335, had higher levels of resistance. Notably, REL11335 was the clone used in Lamrabet et al. (29), where susceptibility of Ara-2 to a range of antibiotics did not visibly differ from the other populations. Overall, the previous work, plus our new findings, suggests that Ara-2 has an even greater amount of phenotypic diversity than previously appreciated.

These results also highlight the potential importance of sampling effects when a single clone is isolated as the representative of a population. If, by chance, that clone is different from the rest of the population, the results could be misleading. This is especially likely in genetically diverse populations, such as those with a long evolutionary history (50) or those evolving in complex environments (51). Such sampling effects may be of particular concern for the measurement of antibiotic resistance because the standard protocol is to measure the minimum inhibitory concentration of one or a few isolated clones, including in applied clinical laboratory settings where polymicrobial infections may be common (52, 53). If a more resistant strain is present but not dominant in an infection, that resistance may be missed during MIC testing. However, antibiotic treatment would be less effective in removing the resistant subpopulation, favoring an increase in the frequency of resistant bacteria.

Although selection under elemental limitation did not affect intrinsic resistance in our experiment, our results highlight the potential for antibiotic susceptibility to vary due to evolutionary history. Furthermore, in this experiment, we focused only on the intrinsic resistance of strains evolved in the absence of antibiotics. Given the widespread nature of elemental limitation, our next experiments will also test whether elemental limitation affects the evolutionary response to direct antibiotic exposure. The costs and benefits of resistance mutations may well differ between limitation environments. For example, expression of outer membrane porins varies dramatically between carbon and nitrogen limitation (54, 55), and the same porins also evolve under antibiotic selection (49), suggesting strong potential for elemental limitation to affect the evolution of antibiotic resistance.

## Data Availability

All data and R scripts are available at Dryad: https://doi.org/10.5061/dryad.1zcrjdg68.

## SUPPLEMENTAL MATERIAL

Supplemental material msphere.00538-25-s0001.docx Supplemental text, Figure S1, and Tables S1 and S2.

ASM does not own the copyrights to Supplemental Material that may be linked to, or accessed through, an article. The authors have granted ASM a non-exclusive, world-wide license to publish the Supplemental Material files. Please contact the corresponding author directly for reuse.
