# Supplementary material for: Antibiotic susceptibility of Escherichia coli is affected by evolutionary history but not by history of elemental limitation
Source: mSphere. 2026 Mar 23;11(4):e00538-25. doi: 10.1128/msphere.00538-25 (PMC13123722; doi:10.1128/msphere.00538-25)
Supplement: Supplemental material — Supplemental text, Figure S1, and Tables S1 and S2. [file msphere.00538-25-s0001.docx]

**Supplemental text:**

Clones within the Ara-2 population are known to vary in their ability to grow on TA plates (1). We began our experiments with a clone isolated from Ara-2 that did exhibit growth on TA plates following growth in carbon-limited media. However, we found that this clone and its evolved derivatives did not reliably form colonies on TA plates following growth under nitrogen limitation. Thus, we were able to directly measure fitness for the Ara-2 clone and its derivatives under carbon-limitation, but not under nitrogen limitation.

However, we were able to infer indirectly that the clones derived from Ara-2 gained fitness under nitrogen limitation. During our competitions, the bacteria in the competition flask as a whole can undergo approximately 6.67 generations, increasing from roughly 5 x 10^5^ cfu/ml to the carrying capacity of roughly 5 x 10^7^ cfu/ml. Therefore, if one competitor undergoes more generations of growth, the other competitor must necessarily undergo fewer generations (assuming the strains are largely competing for the same resources). In our competitions specifically, we measured the growth of the common competitor (REL607) when grown with the 50,000 generation Ara-2 clone and when grown with the clones from the two populations that evolved for 2,000 generations under nitrogen limitation. The common competitor underwent more generations of growth when it competed against the ancestral clone than when it competed against the evolved clones from those two populations (Table S2). This strongly suggests that the evolved clones were more fit than the original Ara-2 clone under nitrogen limitation.

Reference:

1. Wiser MJ, Ribeck N, Lenski RE. 2013. Long-term dynamics of adaptation in asexual populations. Science 342:1364-1367.


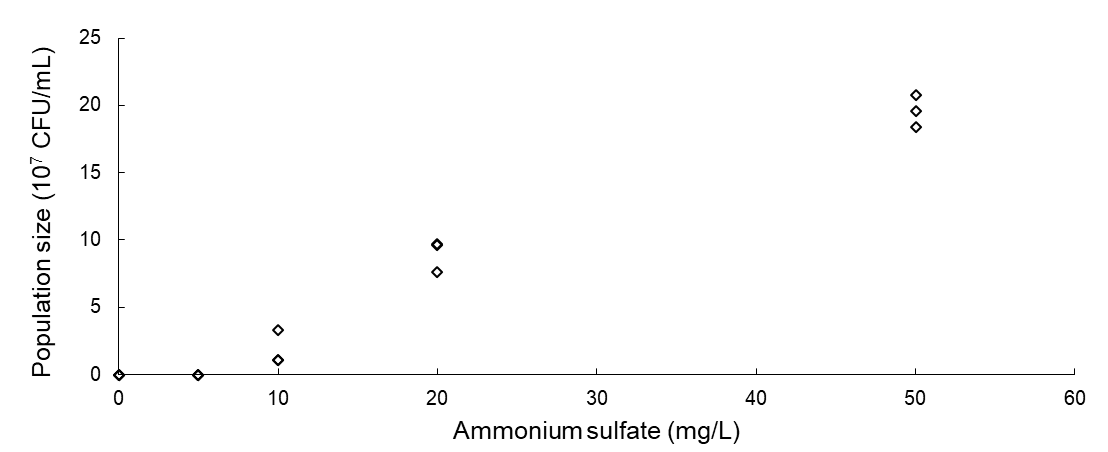


**Figure S1:** In Davis Mingioli minimal media with 250 mg/L glucose, population size increased with additional ammonium sulfate, indicating that population size is limited by nitrogen availability in this environment. Note that the media is not sulfur limited as it also contains magnesium sulfate. Population sizes in 20 mg/L ammonium sulfate media were collected on a different date than those for the other media formulations.

| **Medium** | **0-generation clone** | **1000-generation clone** |
| --- | --- | --- |
| Carbon-limited | REL606 | CBTn288 |
| Carbon-limited | REL606 | CBTn300 |
| Carbon-limited | REL11330 (Ara-1) | CBTn290 |
| Carbon-limited | REL11330 (Ara-1) | CBTn302 |
| Carbon-limited | REL11333 (Ara-2) | CBTn292 |
| Carbon-limited | REL11333 (Ara-2) | CBTn304 |
| Carbon-limited | REL11336 (Ara-4) | CBTn294 |
| Carbon-limited | REL11336 (Ara-4) | CBTn306 |
| Carbon-limited | REL11339 (Ara-5) | CBTn296 |
| Carbon-limited | REL11339 (Ara-5) | CBTn308 |
| Carbon-limited | REL11389 (Ara-6) | CBTn298 |
| Carbon-limited | REL11389 (Ara-6) | CBTn310 |
| Nitrogen-limited | REL606 | CBTn312 |
| Nitrogen-limited | REL606 | CBTn276 |
| Nitrogen-limited | REL11330 (Ara-1) | CBTn266 |
| Nitrogen-limited | REL11330 (Ara-1) | CBTn278 |
| Nitrogen-limited | REL11333 (Ara-2) | CBTn268 |
| Nitrogen-limited | REL11333 (Ara-2) | CBTn280 |
| Nitrogen-limited | REL11336 (Ara-4) | CBTn270 |
| Nitrogen-limited | REL11336 (Ara-4) | CBTn282 |
| Nitrogen-limited | REL11339 (Ara-5) | CBTn272 |
| Nitrogen-limited | REL11339 (Ara-5) | CBTn284 |
| Nitrogen-limited | REL11389 (Ara-6) | CBTn274 |
| Nitrogen-limited | REL11389 (Ara-6) | CBTn286 |

**Table S1:** List of the ancestral and evolved clones for each population.

|  | **Generations of growth of REL607** | |
| --- | --- | --- |
| **Population of evolved clone** | **Versus 50,000 generation Ara-2 clone** | **Versus Ara-2 clone evolved under nitrogen limitation** |
| Population A | 6.4 | 4.8 |
|  | 7.0 | 6.0 |
|  | 6.8 | 6.3 |
| Population B | 7.2 | 6.0 |
|  | 7.0 | 5.5 |
|  | 7.4 | 6.2 |

**Table S2**: Under nitrogen limitation, the common competitor (REL607) underwent more generations of growth when competing against the Ara-2 50,000 generation clone (REL11333) than when competing against derived clones that had evolved under nitrogen limitation (CBTn268 and CBTn280). These data were collected as part of the set of competition experiments depicted in Figure 2.
